# Supplementary material for: Signs of criticality in social explosions
Source: Sci Rep. 2024 Feb 8;14:3263. doi: 10.1038/s41598-024-53657-1 (PMC10853521; doi:10.1038/s41598-024-53657-1)
Supplement: Supplementary file 1 — Supplementary Information. [file 41598_2024_53657_MOESM1_ESM.pdf]

Signs of criticality in social explosions: Supplementary information

Mariano G. Beiró
CONICET – Universidad de Buenos Aires. INTECIN, Argentina, and Universidad de Buenos Aires, Facultad de Ingeniería, Argentina.

Ning Ning Chung
Centre for University Core, Singapore University of Social Sciences, Singapore 599494.

Lock Yue Chew
School of Physical & Mathematical Sciences, Nanyang Technological University, Singapore 637371, and Data Science & Artificial Intelligence Research Centre, Nanyang Technological University, Singapore 639798.

Yérali Gandica*
Laboratoire de Physique Théorique et Modélisation, UMR-8089 CNRS, CY Cergy Paris Université, France, Universidad Internacional de Valencia (VIU), E-46 002, Valencia, Spain. and
**ygandica@gmail.com*

I. POWER LAW EXPONENTS FOR THE HASHTAGS FREQUENCY DISTRIBUTION IN ALL THE PERIODS

Exponents fitted for the power law distribution of the hashtags' frequencies, for the four data-sets and the three time periods ('before', 'during', and 'after' the event), and also for the whole period (i.e., the concatenation of the three periods).

TABLE I. Discrete power law exponents for the frequency distribution of hashtags, fitted by max-likelihood for each event and time period. Standard errors are reported in parenthesis in units of the least significant digit. We show results by counting the hashtags every time a user posted it ('Hasht', above), and only once ('User', below), irrespective of how many times it was used by the same user. The x_{min} parameters were also fitted by max-likelihood, and all fits passed the Kolmogorov-Smirnov goodness-of-fit test at p=0.05.

**Level**	**Dataset**	**Same time**				**Same nº of hashtag usages**			
		Before	*During*	*After*	*All*	*Before*	*During*	*After*	*All*
**Tweet**	noaltarifazo/ruidazonac..	1.957(11)	1.943(11)	1.964(11)	1.904(7)	1.955(11)	1.943(11)	1.937(9)	1.900(7)
	9n/9ngranmarchaporlaj..	1.822(11)	1.760(10)	1.755(10)	1.768(7)	1.818(9)	1.760(10)	1.755(9)	1.768(6)
	15m	1.960(46)	1.825(23)	1.931(23)	1.861(16)	1.793(20)	1.825(23)	1.941(25)	1.816(14)
	Charlie Hebdo	2.036(16)	1.988(14)	2.031(17)	1.976(10)	2.054(13)	1.988(14)	2.109(12)	2.015(8)
**User**	noaltarifazo/ruidazonac..	1.994(12)	1.989(11)	2.028(12)	1.969(7)	1.989(12)	1.989(11)	2.006(10)	1.966(7)
	9n/9ngranmarchaporlaj..	1.788(10)	1.758(10)	1.757(10)	1.772(7)	1.822(9)	1.758(10)	1.755(9)	1.761(6)
	15m	2.125(54)	1.870(24)	1.947(23)	1.881(17)	1.840(20)	1.870(24)	1.960(25)	1.886(15)
	Charlie Hebdo	2.052(16)	1.974(14)	1.958(16)	2.008(10)	2.028(13)	1.974(14)	2.323(15)	2.071(9)
